# Autophagy Attenuation Hampers Progesterone Synthesis during the Development of Pregnant Corpus Luteum

**DOI:** 10.3390/cells9010071

**Published:** 2019-12-27

**Authors:** Zonghao Tang, Zhenghong Zhang, Hong Zhang, Yuhua Wang, Yan Zhang, Jiuhua Zhao, Hongqin Yang, Zhengchao Wang

**Affiliations:** 1 Provincial Key Laboratory for Developmental Biology and Neurosciences, Key Laboratory of Optoelectronic Science and Technology for Medicine of Ministry of Education, College of Life Sciences, Fujian Normal University, Fuzhou 350007, China; tangzonghao@163.com (Z.T.); zhangzh@fjnu.edu.cn (Z.Z.); ZhHong0898@163.com (H.Z.); yuhwang@fjnu.edu.cn (Y.W.); yanzhang970118@sina.com (Y.Z.); zhao9hua@126.com (J.Z.); 2 Drug Discovery Research Center, Southwest Medical University, Luzhou 646000, China

**Keywords:** autophagy, lipid droplet, corpus luteum, progesterone, pregnancy

## Abstract

**Simple Summary:**

The present study demonstrates that induction of autophagy-related proteins in corpus luteum is regulated by Akt/mTOR signaling and autophagy may exert influences on progesterone production by controlling the pool of lipid droplets in luteal cells during the luteal development of pregnant rats. Furthermore, mitophagy-related proteins were also induced during the initiation of luteal regression in pregnant rats, which may play an essential role in the maintenance of mitochondrial homeostasis. These findings will shed light on the role of autophagy during the luteal development of pregnant ovaries in vivo in mammals.

**Abstract:**

The contribution of autophagy to catabolic balance has been well-established in various types of cells, whereas the involvement of autophagy in progesterone synthesis during rat pregnancy still remains unknown. Therefore, the present study was designed to evaluate the role of autophagy in progesterone production during the luteal development of pregnant rats. The results showed autophagy-related proteins was maintained at a low level on day 10 after pregnancy, significantly induced on day 16 and subsided to a relative low level on day 21, which was consistent with the changes of serum progesterone levels. The findings further indicated the contribution of autophagy to progesterone production was regulated by inactivation of Akt/mTOR signaling during the luteal development of pregnant rats in in vivo and in vitro experiments. Further investigations revealed autophagy may be involved in the surge of progesterone production in pregnant rats, as inhibition of autophagy by 3-MA compromised serum progesterone levels. Furthermore, 3-MA treatment also leveled down the number of lipid droplets in luteal cells, implying that autophagy may affect the production of progesterone by manipulating the formation of lipid droplets in luteal cells. In addition, the results suggested that mitophagy was mobilized during the primary stage of luteolysis and inhibition of autophagy promoted the increase of redundant mitochondrial and cytoplasmic cytochrome C in luteal cells of pregnant rats. Taken together, the present study indicated that autophagy-related proteins were induced by the inactivation of Akt/mTOR signaling and then contributed to the progesterone production possibly by affecting the formation of intracellular lipid droplets during the luteal development of pregnant rats. To our knowledge, this will provide a new insight into the important mechanism of autophagy regulating progesterone production in ovaries of pregnant mammals.

## 1. Introduction

Autophagy is an evolutionarily conserved cellular catabolic activity by degrading unessential or redundant parts of itself for recycling of building blocks, ensuring homeostasis of metabolism under stresses and contributing to survival of cells [1]. During this program, several essential proteins are involved in induction or degradation of autophagosomes, including Beclin1—a scaffold protein for autophagosome initiation, LC-3—a protein involved in autophagosome formation, and LAMP2—a protein involved in lysosome regulation. The induction of autophagy is highly regulated, of which the role is tightly associated with many aspects of cell activities, such as cell proliferation [2], metabolism [3], and hormone production [4]. Autophagy dysregulation is widely acknowledged as a causative factor for various diseases, such as cancer [5] and muscle atrophy [6]. In mammals, studies have revealed that the level of autophagy in steroidogenic cells is higher than in other types of cells and aberrant regulation of autophagy is related to turbulent steroidogenesis [7]. Likewise, steroids also exert influences on autophagy induction, suggesting sophisticated regulatory mechanisms between autophagy induction and steroid synthesis [4].

The corpus luteum (CL) is an ephemeral hormonal gland evolving from remains of ovarian follicles after ovulation in a mammalian ovary, which functions as a biological alarm clock of a female estrous cycle and a prominent controller of a pregnant program. The main function of the CL is to produce progesterone, so as to ensure the development of embryo during pregnancy. The deficiency of progesterone not only contributes to abnormal embryos development but also impairs menstrual cycles in mammals [8]. In human, the CL of a menstrual cycle secretes up to 25 mg of progesterone per day [9], and cholesterol is recognized as a raw material for progesterone production in mammals [10]. Since progesterone is derived from cholesterol, the harmonization of cholesterol uptake, storage, and metabolism makes up an integral process of a steroidogenic program. In rats, the level of progesterone begins to increase on day 10 of pregnancy, reaches its maximum on day 16 and thereafter decreases to a basal level on day 21. The available evidence indicated that plasma lipoprotein is the major source of cholesterol uptake for steroidogenesis in humans or rats [8]. Autophagy has been shown to be involved in lipid metabolism, through a so-called “lipophagy” process to provide cells with triglycerides and cholesterol. Interestingly, autophagy also participates in lipid storage [11] and contributes to accumulation of lipid droplets in many species, including mouse [12], yeast [13], elegans [14], and *Drosophila* [15]. Accordingly, these findings suggested a ubiquitous regulatory role of autophagy in lipid storage. Compared with other cell types, steroidogenic cells demand a large amount of cholesterol for steroid synthesis, whereas the involvement of autophagy and the mechanism underlying its regulation still remain largely unknown.

In steroidogenic cells, mitochondria is responsible for progesterone synthesis, whereas the hyperactivation of mitochondria is also associated with the release of its byproduct, Reactive oxygen species—ROS [16]. Compelling evidences have indicated that autophagy exerts influences on controlling mitochondrial quality by degrading redundant or impaired mitochondria, ensuring the homeostasis of cell physiologies [17]. However, whether autophagy is involved in mitochondrial quality control during the luteal development of pregnant rats still remains to be clarified. In addition, our previous studies have demonstrated the expression changes of autophagy during all three developmental phases of the CL in pregnant rats and found a significant increase of autophagic expressions during the late luteal phase (LLP) in the ovaries of pregnant rats [18,19], but the molecular mechanism regulating this change still remains unknown. Therefore, the present study was designed to investigate the physiological contribution and the underlying mechanism of autophagy to progesterone production during the luteal development of pregnant rats.

## 2. Materials and Methods

### 2.1. Animals

A total of 80 female Sprague-Dawley (SD) rats (about 250 g body weight) and 18 male SD rats (about 250 g body weight) were purchased from Wushi Experimental Animal Supply Co. Ltd. (Fuzhou, China). The animals were maintained under a 14 h light/10 h dark schedule with continuous supplies of chow and water. The study was conducted in accordance with the Declaration of Helsinki, and the experimental protocol was approved by the Institutional Animal Care and Use Committee and the Ethics Committee on Animal Experimentation, Fujian Normal University (project identification code: IACUC-20170020).

### 2.2. Experimental Design

The rats were allowed to accommodate for 1–2 weeks prior to mating with males. Previously unmated female rats (three per cage) were mated with an unvasectomized male (one per cage) and were examined every morning for the presence of a vaginal plug. Day 1 of pregnancy was defined as the day, at which a vaginal plug was recovered. The pregnant females were removed and used in subsequent experiments.

In order to determine possible roles of autophagy, 3-MA (an autophagy inhibitor, i.p. (intraperitoneal) 15 mg/kg body weight, Sigma-Aldrich, St. Louis, MO, USA) was injected according to the method described by Choi et al. [20]. Briefly, 3-MA was dissolved in sterile saline, and then pregnant rats were consecutively treated for 5 days (i.p) before samples collection; saline was served as the control/vehicle. All pregnant rats were executed at three designed time points, including day 10 when progesterone was surging, day 16 when the CL status or functions at noon required the full-load operation of mitochondria, and day 21 when the circulatory progesterone level dramatically decreased. The ovarian samples were harvested and prepared for the following experiments. Briefly, one ovary from each rat was fixed in 4% paraformaldehyde for immunohistochemistry staining or TEM observation, and the other one was immediately suffered to the isolation of luteal cells for primary culture or snap-frozen for the remaining experiments.

### 2.3. Immunohistochemistry of LC-3 and LAMP-2

After fixation, ovaries from each rat were embedded in paraffin, and 5 μm sections were cut and mounted on slides. The sections were then processed for immunohistochemical analysis with anti-LC-3 antibody (1:500 *v*/*v*, Abcam, Cambridge, MA, USA) and anti-LAMP-2 antibody (1:200 *v*/*v* dilution, Protein Tech Group, Wuhan, China). The sections were incubated at 4 °C overnight with a primary antibody. The immunoreactivity of these specific proteins was visualized by the Elite ABC kit (BioGenex, San Ramon, CA, USA). Then, the sections were counter-stained with hematoxylin and mounted with cover slips to identify the structure and types of cells in rat ovaries. The negative control used a serum (Boster Biological Technology, Wuhan China) instead of a primary antibody, and these slides were used for histological examination.

### 2.4. Determination of Progesterone Levels

The concentrations of the serum and the medium progesterone were examined using a rat progesterone ELISA kit (Xiamen Huijia Biological Technology Company, Xiamen, China), according to the manufacturer’s protocols. Briefly, the reagents were prepared according to the provided instruction. After that, the standard progesterone and samples were added into each well. Then, the samples were incubated with each reagent for designated times. After washing, the result was acquired by a microplate reader.

### 2.5. Western Blot Analysis

The expressions of the interested proteins were examined in both in vivo and in vitro samples. CLs wwere separated from ovaries under a dissecting microscope with great care. Briefly, the intact sections of ovaries were recognized as CLs, while the semitransparent one was recognized as follicle because the follicle was hollow in the inside. The isolated CLs were homogenized, and the total lysates were extracted by using an ice-cold RIPA buffer with supplemented protease inhibitors (protease inhibitor cocktail, Beyotime Institute of Biotechnology, Haimen, China). Mitochondria from different samples were removed for cytochrome C examination by using a commercial kit according to the instruction of the manufacturer (Beyotime Institute of Biotechnology, Haimen, China). The lysates of cell samples were also prepared similarly. Protein concentrations were determined by a Bio-Rad protein assay (Bio-Rad, Hercules, CA, USA) with bovine serum albumin standards. Twenty micrograms of protein samples were subjected to SDS-PAGE gel electrophoresis and then electrophoretically transferred onto a polyvinylidene difluoride (PVDF; Pall Life Sciences, Port Washington, NY, USA) membrane. The nonspecific binding was blocked by 5% skimmed milk, and the membranes were thereafter incubated overnight in the presence of primary antibodies (Supplementary Table S1). After washing with TBST, the membranes were incubated in horseradish peroxidase-conjugated goat antirabbit or mouse IgG (1:1000 dilution, Beyotime Institute of Biotechnology, Haimen, China) for 1 h at room temperature. The bands were visualized using enhanced chemiluminscence (ECL) reagents (Beyotime Institute of Biotechnology, Haimen, China). The blots were quantified using ImageJ 1.49 software (the National Institutes of Health, Bethesda, MD, USA).

### 2.6. Oil Red O Staining

Ovarian samples were fixed, dehydrated and then embedded in an optimal cutting temperature (OCT) compound. After section, frozen sections (5 μm) were allowed to be air-dried for 24 h and then washed in PBS for three times to remove the OCT compound. The sections were stained with freshly prepared 0.15% Oil Red O for 10 min after incubating in 60% isopropyl alcohol for 5 min. Then, the sections were washed in 60% isopropyl alcohol to remove unspecific attachment, counterstained with hematoxylin and mounted with glycerol jelly.

### 2.7. TEM

CL tissues were collected from ovaries of pregnant rats with or without 3-MA treatment. Specimens for TEM were prepared and fixed with 2.5% glutaraldehyde (P1126, Solarbio) in PBS (4 °C, pH 7.4, 0.1 M) for 24 h. The samples were then postfixed with 1% OsO4 (Ted Pella) for 1.5 h and stained with 3% aqueous uranyl acetate for 1 h after washing. After that, the samples were dehydrated by graded alcohol series, embedded in Araldite (90529-77-4, SPI-CHEM), sectioned with a thickness of approximately 60 nm and mounted on Formvar-coated grids (01700-F, Ted Pella). The ultrathin sections were contrasted with 0.3% lead citrate stainning, examined and photographed under a transmission electron microscope (JEM-2100, JEOL, Tokyo, Japan).

### 2.8. Isolation and Culture of Luteal Cells

Luteal cells were isolated by slight modifications of previously described methods [21]. Briefly, rat ovaries were harvested on day 16 of pregnancy. Bursa and fat tissues were stripped, and then luteal cells were isolated with a needle under a dissecting microscope. The isolated tissue was washed twice with medium and incubated at 37 °C for about 1 h in 10 mL of the collagenase type 1 solution (1 mg/mL in DMEM/F12). The dissociated luteal cells were collected, after the DMEM/F12 medium with 10% FBS was added to stop further dissociation, and the undigested tissue was discarded. The supernatant-containing dispersed luteal cells were thereafter centrifuged (250 *g*) for 10 min to yield a luteal cell pellet. Trypan blue was used to determine cell viability, and then the collected cells were found to have a viability of >90%. Luteal cells were cultured in the DMEM/F12 medium at 37 °C in a humidified atmosphere composed of 95% air and 5% CO_2_. To investigate the mechanism of autophagy induction, luteal cells were treated with rapamycin (100 nM) for 0, 30, 60, and 120 min. The effect of autophagy on progesterone synthesis was evaluated by rapamycin and 3-MA (5 μM) treatment. For progesterone measurements, luteal cells (1 × 105 cells/well) were plated into 24-well culture plates in 1 mL of a 1:1 *v*/*v* mixture of DMEM/F-12 medium containing 15 mM HEPES (pH 7.4), 100 U/mL penicillin G, and 100 mg/mL streptomycin (Hyclone, Logan, UT, USA). Likewise, luteal cells were transfected with scramble or Beclin1 small interfering RNA (siRNA), and then, the secretion of progesterone was detected by an ELISA kit (Xiamen Huijia Biological Technology Company, Xiamen, China), according to the manufacturer’s protocols.

### 2.9. Immunofluorescence Staining

After treatment, luteal cells were washed with PBS, then fixed with 4% paraformaldehyde and blocked with 0.1% BSA for 1 h. Cells were incubated with the LC-3 primary antibody (1:500 *v*/*v*) in PBS and reacted with an Alexa 488-conjugated secondary antibody (1:500 *v*/*v* dilution; Thermofisher, A-21206). Finally, slides were mounted in mounting media (Beyotime Institute of Biotechnology, Haimen, China), and images were captured with a confocal laser scanning microscope (Carl Zeiss, Göttingen, Germany). The punctuates of cells were calculated by ImageJ (NIH, Bethesda, MD, USA).

### 2.10. siRNA Assay

To knockdown the Beclin 1 expression, the siRNAs-targeting Beclin1 and nontargeting scramble siRNA were synthesized by GenePharma, China. The sequences of siRNAs were as follows: Beclin 1 siRNA1, sense, 5′-CUC AGG AGA GGA GCC AUU UTT-3′, antisense, 5′-AAA UGG CUC CUC UCC UGA GTT-3′ and negative control siRNA, sense, 5′-UUC UCC GAA CGU GUC ACG UTT-3′, antisense, 5′-ACG UGA CAC GUU CGG AGA ATT-3′. Luteal cells were seeded at 2 × 105 per well in 6-well plates overnight, and the transfection was performed, when the cells reached a 70% confluence. During transfection, siRNA duplexes (50 pmol) were transfected into the target cell populations using the Lipofectamine 3000 Reagent (Invitrogen, Carlsbad, CA, USA) according to the manufacturer’s instructions. Culture media were collected for progesterone measurements 48 h after transfection, and cell proteins were extracted using RIPA for assessing protein expression levels by western blotting.

### 2.11. Statistical Analysis

All data were presented as means ± SE (standard error). The significant differences within or between treatment groups were evaluated by ANOVA, followed by the Tukey’s multiple range test. Statistical analysis was conducted using SPSS version 20 software, and *p* < 0.05 was recognized as significantly different.

## 3. Results

### 3.1. Autophagy-Related Proteins Were Significantly Induced during the Surge of Progesterone Secretion in Pregnant Rats

**Figure 1:**
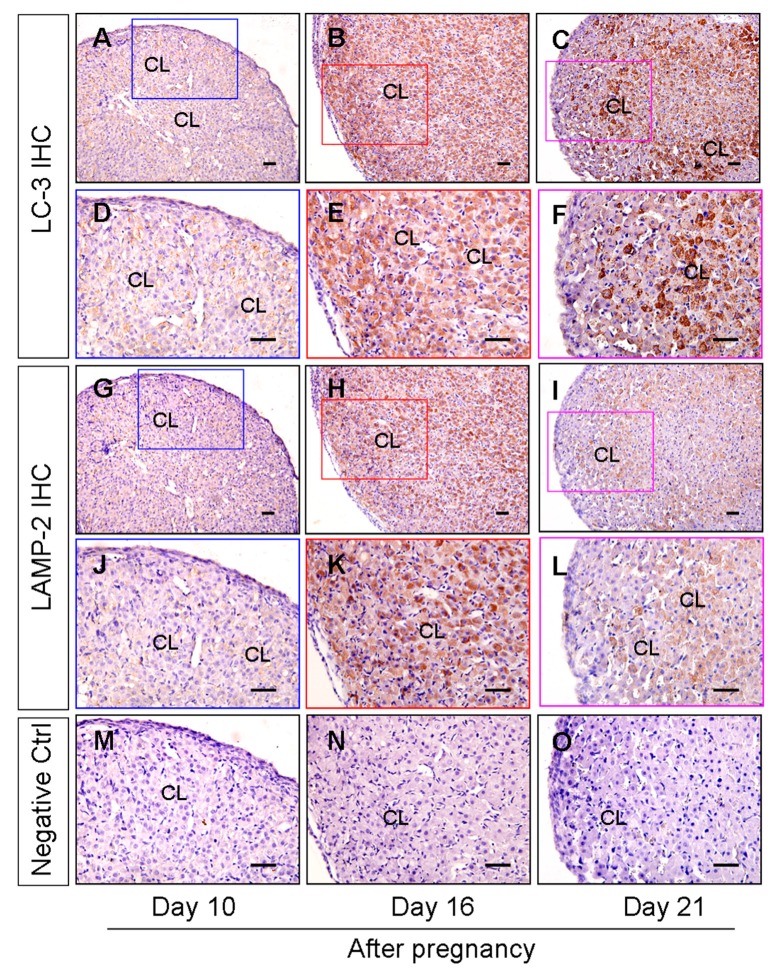
Expression and localization of LC-3 and LAMP-2 during the luteal development in the ovaries of pregnant rats. LC-3 (**A**–**F**) and LAMP-2 (**G**–**L**) immunohistochemical signals appear brown, and the counterstaining background appears blue in color. Negative controls remained unstained, lacking a primary antibody instead of a serum (**M**–**O**). (**A**,**D**,**G**,**J**,**M**) are results on day 10 after pregnancy; (**B**,**E**,**H**,**K**,**N**) are results on day 16 after pregnancy; (**C**,**F**,**I**,**L**,**O**) are results on day 21 after pregnancy. CL: corpus luteum. Scale bar = 100 µm.

**Figure 2:**
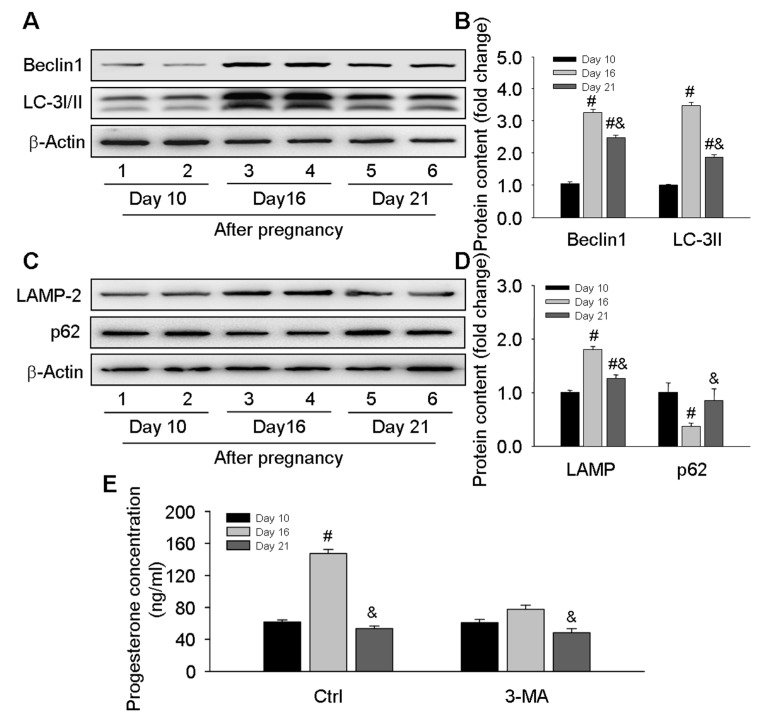
Changes of ovarian-autophagy-related protein levels and serum progesterone concentrations during the luteal development of pregnant rats: (**A**) representative western blot analyses depicting the protein levels of Beclin1 and LC-3; (**B**) summarized intensities of Beclin1 and LC-3II blots normalized to the control; (**C**) representative western blot analyses depicting the protein levels of LAMP-2 and p62; (**D**) summarized intensities of LAMP-2 and p62 blots normalized to the control; (**E**) serum progesterone levels. Each value represents the mean ± SE, n = 6. ANOVA was used to analyze the data. #: *p* < 0.05 vs. *p* on day 10, &: *p* < 0.05 vs. *p* on day 16. 3-MA: an autophagy inhibitor.

To clarify the involvement of autophagy in the pregnant CL development during the middle to late stages, the autophagy level was evaluated in CLs at different time points of pregnancy. As LC-3 and LAMP2 are two marker proteins for autophagosome and lysosome evaluation, both of them could be induced during autophagy induction. We here located both proteins by immunohistochemical staining. The results of LC-3 and LAMP-2 immunostaining demonstrated the obvious induction of autophagy and a significantly increased number of lysosomes in the CLs on day 16 after pregnancy followed by a decrease on day 21 (Figure 1). The expression levels of autophagic marker proteins including Beclin1, LC-3, and LAMP-2 were further detected by western blotting (Figure 2A–D), and it was found these proteins were significantly increased on day 16 and then compromised on day 21 but still maintained at a high level compared with that on day 10 (Figure 2A–D), which corroborated the immunostaining findings of autophagy during the luteal development of pregnant rats (Figure 1). Notably, although the expression changes of p62 was consistent with the tendency of autophagy induction on day 10 and day 16, it is not the case on day 21, as the level of p62 was even higher than on day 10, which may indicate a decrease of autophagy flux (Figure 2C,D). Interestingly, the variation of autophagy levels was synchronized with the changes of serum progesterone levels (Figure 2E), which were blocked by an autophagy inhibitor 3-MA (Figure 2E), further indicating the involvement of autophagy during the luteal development of pregnant rats.

### 3.2. Akt/mTOR Signaling Was Involved in the Induction of Autophagy-Related Proteins during the Luteal Development of Pregnancy

**Figure 3:**
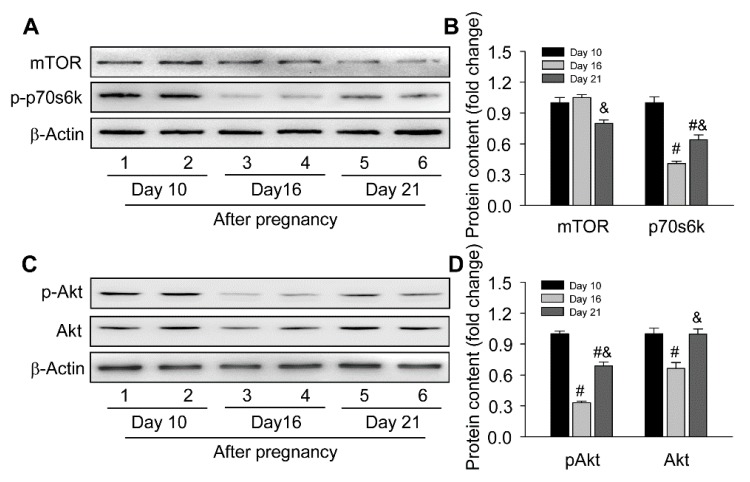
Expressions changes of Akt/mTOR signaling during the luteal development in ovaries of pregnant rats: (**A**) representative western blotting analyses depicting the protein levels of mTOR and p-p70s6k; (**B**) summarized intensities of mTOR and p-p70s6k blots normalized to the control; (**C**) representative western blotting analyses depicting the protein levels of p-Akt and Akt; (**D**) summarized intensities of p-Akt and Akt blots normalized to the control. Each value represents the mean ± SE, n = 6. ANOVA was used to analyze the data. #: *p* < 0.05 vs. *p* on day 10, &: *p* < 0.05 vs. *p* on day 16.

To further understand the molecular mechanism regulating autophagy induction, the present study focused on Akt/mTOR signaling, which is the master regulator of autophagy in mammalian cells [22], including steroid-producing cells [7]. The present results found the activation of mTOR singling was inhibited by downregulated p-p70s6k on day 16 and day 21 of pregnancy (Figure 3A,B). Thereafter, the upstream factor of mTOR, the Akt activation, was further analyzed, and it was shown the expressions of Akt and pAkt significantly decreased on day 16 (Figure 3C,D), implying Akt/mTOR signaling was highly related with the induction of autophagy-related proteins during the luteal development of pregnancy.

**Figure 4:**
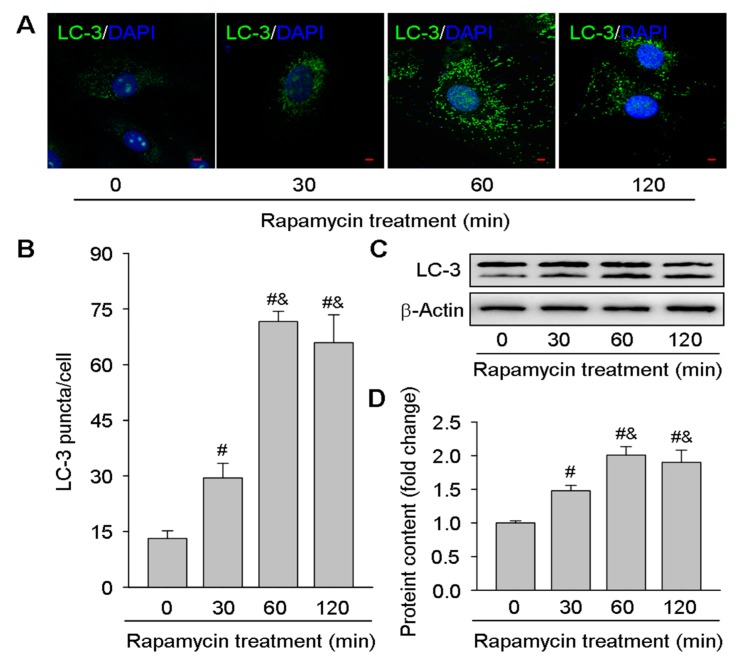
Effects of rapamycin on LC-3II expressions in luteal cells cultured in vitro. Luteal cells were isolated from rat ovaries on day 16 of pregnancy and then cultured with rapamycin (100 nM) for 0, 30, 60, and 120 min: (**A**) representative immunofluorescent pictures depicting the protein levels of LC-3; (**B**) summarized puncta of LC-3 immunofluorescence; (**C**) representative western blotting analyses depicting the protein levels of LC-3; (**D**) summarized intensities of LC-3II blots normalized to the control. Each value represents the mean ± SE, n = 6. ANOVA was used to analyze the data. #: *p* < 0.05 vs. *p* on day 10, &: *p* < 0.05 vs. *p* on day 16. Rapamycin: an mTOR inhibitor, LC-3: an autophagy marker protein. Scale bar = 20 µm.

For further confirming the results of in vivo experiments, the present study also isolated luteal cells from pregnant rats CLs on day 16 and then treated with an mTOR inhibitor rapamycin (Figure 4). The immunofluorescence staining for LC-3 suggested the autophagy was induced by rapamycin in a time-dependent manner in luteal cells (Figure 4A,B), which was further verified by the upregulated expressions of LC-3II after rapamycin treatment (Figure 4C,D). These findings further indicated the role of mTOR signaling in autophagy regulation during the luteal development of pregnancy.

### 3.3. Regulatory Role of Autophagy in Progesterone Production in Luteal Cells

**Figure 5:**
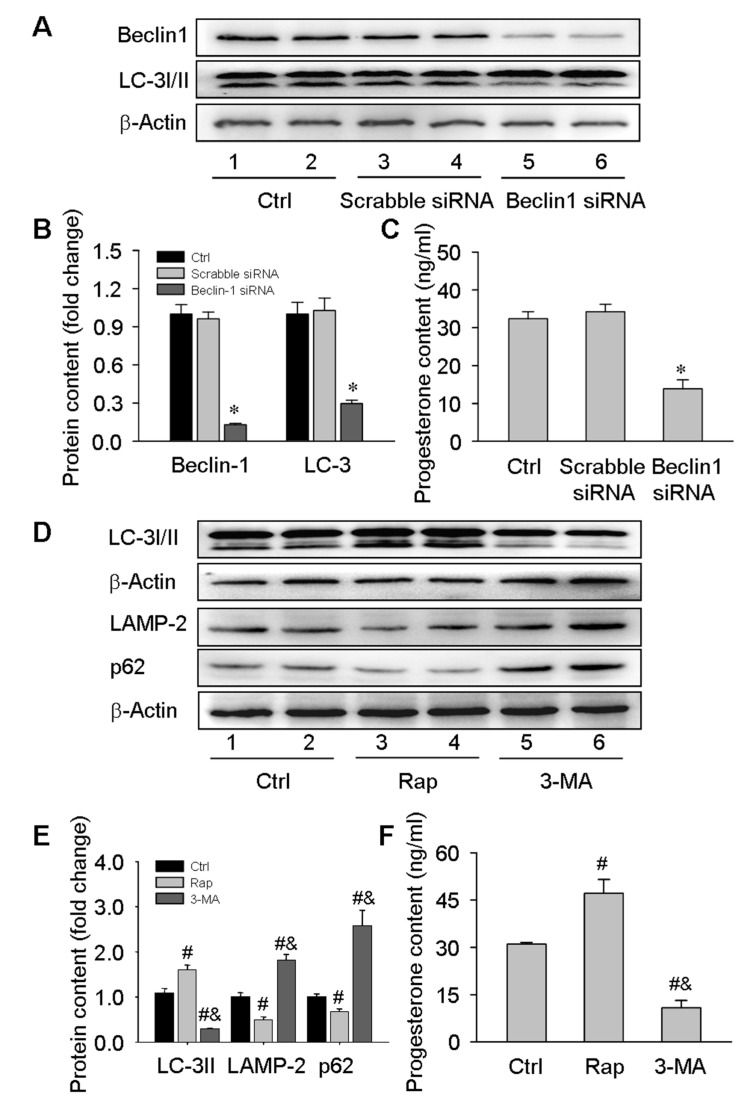
Contribution of autophagy to progesterone production in luteal cells cultured in vitro. Luteal cells were isolated from rat ovaries on day 16 of pregnancy and then cultured with Beclin1 siRNA transfection or Rap/3-MA treatment: (**A**) representative western blot analyses depicting the protein levels of Beclin1 and LC-3 in luteal cells transfected with scrabble or Beclin1 siRNA. The luteal cells were plated on 6-well plates one day before transfection, and the transfection was performed at a 70% confluence. The protein contents were detected 48 h after transfection; (**B**): summarized intensities of Beclin1 and LC-3II blots normalized to the control; (**C**) serum progesterone levels; (**D**) representative western blot analyses depicting the protein levels of LC-3I/II, LAMP-2, and p62 in luteal cells treated with Rap or 3-MA. The luteal cells were plated on 6-well plates one day before treatment. The cells were remained as untreated controls or treated with Rap or 3-MA for 24 h; (**E**) summarized intensities of LC-3II,LAMP-2, and p62 blots normalized to the control; (**F**) serum progesterone levels. Each value represents the mean ± SE, n = 6. ANOVA was used to analyze the data. *: *p* < 0.05, #: *p* < 0.05 vs. *p* of Ctrl group, &: *p* < 0.05 vs. *p* of Rap group.

Given the effects of an autophagy inhibitor 3-MA on serum progesterone levels during the luteal development of pregnancy (Figure 2E), the present study transfected luteal cells isolated on day 16 of pregnancy with Beclin1 siRNA or treated these cells with rapamycin and 3-MA. The results showed the secretion of progesterone was obviously diminished by inhibiting autophagy after knockdown of Beclin1 expression with siRNA transfection (Figure 5A–C), which was consistent with the results of 3-MA treatment (Figure 5D–F). Furthermore, the mTOR inhibitor rapamycin promoted progesterone secretion by inducing the expression of autophagy-related proteins (Figure 5D–F). Notably, given that the secretion of progesterone is not an acute program, we here treated luteal cells for 24 h instead of 60 or 120 min according to the investigation previously [23]. These results demonstrated that autophagy may contribute to progesterone production in luteal cells in vivo and in vitro during the luteal development of pregnancy.

### 3.4. Inhibition of Autophagy Compromised the Storage of Lipid Droplets during the Luteal Development of Pregnancy

**Figure 6:**
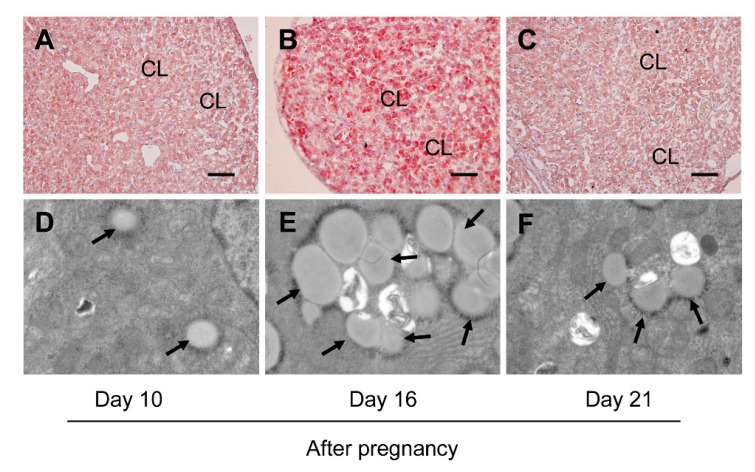
Changes of the storage of lipid droplets in ovaries during the luteal development of pregnant rats: (**A**–**C**) the storage of lipid droplets in CLs on days 10, 16, and 21 of pregnancy by Oil Red O staining, respectively; (**D**–**F**) the storage of lipid droplets in CLs on days 10, 16, and 21 of pregnancy by TEM observation, respectively. The arrows pointed to lipid droplets. CL: corpus luteum. Scale bar = 100 μm.

**Figure 7:**
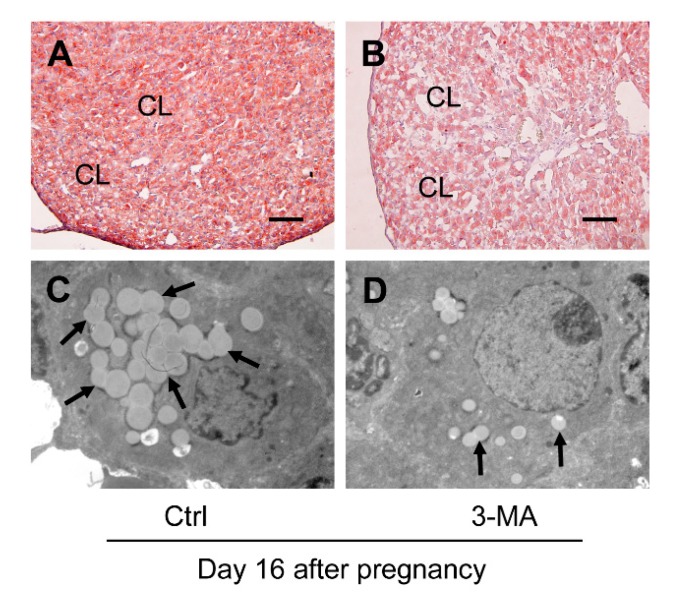
Effects of 3-MA on the storage of lipid droplets in ovaries on day 16 after pregnancy: (**A**,**B**) the storage of lipid droplets in ovaries on day 16 after pregnancy with and without 3-MA treatment by Oil Red O staining; (**C**,**D**) the storage of lipid droplets in ovaries on day 16 after pregnancy with and without 3-MA treatment by TEM observation. The arrow pointed to lipid droplets. CL: corpus luteum. Scale bar = 100 μm.

Furthermore, the present study examined the lipid droplet storage for further clarifying whether autophagy may exert influences on progesterone production by modulating lipid droplet formation, since previous study indicated the increase of lipid droplet storage may be related to progesterone synthesis in luteal cells [24]. Thus, the storage of lipid droplets in CLs was detected on days 10, 16, and 21 of pregnancy by Oil Red O staining and TEM observation. The results suggested the volume of lipid droplets reached the maximum on day 16 (Figure 6B) and exhibited weak staining intensities on both day 10 (Figure 6A) and day 21 (Figure 6C), which was also verified by the results of TEM observation (Figure 6D–F). Consistently, the intensity of Oil Red O staining in the CLs (Figure 7A,B) and the number of lipid droplets (Figure 7C,D) in luteal cells on day 16 of pregnancy were markedly dwindled by 3-MA treatment, implying that autophagy may participate in progesterone production by increasing the storage of lipid droplets during the luteal development of pregnancy.

### 3.5. Inhibition of Autophagy Exacerbated Mitochondrial Homeostasis during the Luteal Development of Pregnancy

**Figure 8:**
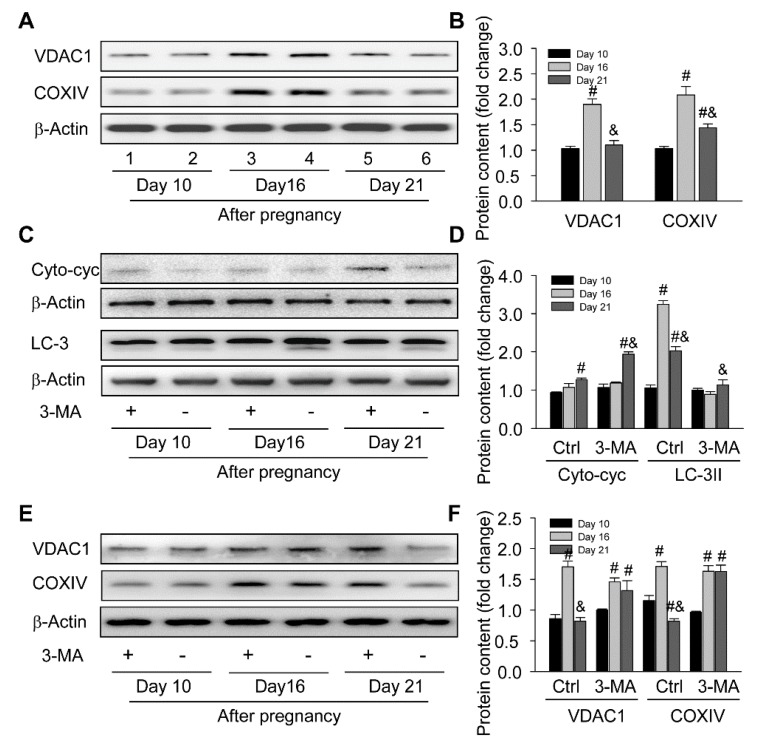
Requirement of autophagy for mitochondria homeostasis in ovaries during the luteal development of pregnant rats: (**A**) representative western blot analyses depicting the protein levels of VDAC1 and COXIV in ovaries during the luteal development of pregnant rats; (**B**) summarized intensities of VDAC1 and COXIV blots normalized to the control; (**C**) representative western blot analyses depicting the protein levels of Cyto-cyc and LC-3 in ovaries with or without 3-MA treatment during the luteal development of pregnant rats; (**D**) summarized intensities of Cyto-cyc and LC-3II blots normalized to the control; (**E**) representative western blot analyses depicting the protein levels of VDAC1 and COXIV in ovaries with or without 3-MA treatment during the luteal development of pregnant rats; (**F**) summarized intensities of VDAC1 and COXIV blots normalized to the control. Each value represents the mean ± SE, n = 6. ANOVA was used to analyze the data. #: *p* < 0.05 vs. *p* on day 10 &: *p* < 0.05 vs. *p* on day 16. 3-MA: an autophagy inhibitor.

Interestingly, the hyperactivity of mitochondria in luteal cells is the perquisite for mass steroid synthesis, which may also hamper the homeostasis of itself and promote the aging of cells [25]. Thus, the present study further evaluated the number of mitochondria by detecting two mitochondrial marker proteins, COXIV localized in an inner mitochondrial membrane and VDAC1 localized in an outer mitochondrial membrane, and then found the number of mitochondria was significantly increased on day 16 and thereafter subsided on day 21 after pregnancy (Figure 8A,B), which was consistent with our above findings.

In order to assess whether autophagy contributed to the quality control of mitochondria during the luteal development of pregnancy, the present study also examined the expressions of autophagy and mitochondrial marker proteins in CLs with or without 3-MA treatment and then found inhibition of autophagy promoted the increase of cytoplasmic cytochrome C (Figure 8C,D) and the accumulation of mitochondria (Figure 8E,F) on day 21 compared with those in the control, while no significant changes in mitochondrial amount were observed either on day 10 or on day 16 after pregnancy (Figure 8E,F), suggesting autophagy may be involved in the quality control of mitochondria by eliminating redundant mitochondria during the functional regression of pregnant CLs, so as to retard the further increase of cytochrome C levels in cytoplasm.

## 4. Discussion

The results of the present study clearly demonstrated that the induction of autophagy is essential for progesterone production in luteal cells in vivo and in vitro, suggesting autophagy may play an important role during the luteal development of pregnant mammals in vivo.

In mammals, highly regulated autophagic programs play versatile roles in ovarian physiologies, including the development of ovarian follicle and CLs [18,19,26,27,28]. Our previous studies have demonstrated the development of CL in pregnant rats included three developmental phases—the early luteal phase (ELP, days 1 to 4 after pregnancy), the middle luteal phase (MLP, days 5 to 6 after pregnancy), and the LLP (days 17 to 21 after pregnancy)—according to the changes of progesterone secretion [28], and we monitored the variation of autophagy-related proteins during these three phases. The autophagy level was dramatically increased during the LLP [18], which may participate in the process of luteal regression through accumulated autophagosomes and excessive apoptosis induced during the luteal development of pregnant rats [19], but the mechanism regulating phase-specific changes of autophagy still remains unclear. Notably, Thomas et al. found the knockout of Beclin-1 resulted in the reduction of progesterone production [27], which indicated autophagy as an important regulator involved in the synthesis of progesterone. Interestingly, they noticed that the lack of Beclin1 also affected the pool of lipid droplets in luteal cells, indicating autophagy is required for maintaining the pool of lipid droplets. Therefore, the present study further investigated the variation, regulation, and physiological contributions of autophagy to progesterone production during this luteal development of pregnant rats.

The ability of progesterone secretion was concomitantly increased and reached its maximum on day 16 during the luteal development of pregnant ovaries in rats [29]. After that, the circulating level of serum progesterone significantly decreased to a basal level on day 21, permitting the occurrence of parturition [29]. Progesterone was endowed with the ability to modulate its own production and oppose functional regression of the CL induced by exogenous agents. In pregnant women, the CL only produced P4 for a limited period of time before the maturation of placenta [30]. Thus, before the establishment of placenta function, the functioning of CL plays an important role for early embryo development. In contrast, rat placenta produced negligible amounts of progesterone, and the CL was responsible for producing steroid throughout gestation [31]. Thus, the CL is essential for the modulation of hormonal balance and for the march of gestation throughout the whole process of rat pregnancy.

Steroid production is an energy-consuming program, which requires orchestration of several organelles and high regulation of cell metabolism. In rats, the CL functioned as a primary plant for progesterone synthesis. On day 10 after rat pregnancy, the sizes of CL and vessel volume began further increased, prepared for the surge of progesterone secretion [32]. On day 16, the level of progesterone was overwhelmingly increased, and the metabolic activity of luteal cells underwent a further upgrade. The significant changes of cellular functions or metabolic status generally enabled the induction of autophagy in mammalian cells, exerting influences on the homeostasis of cells under hypoxia and starvation [3]. Elegantly, this delicately designed mechanism also contributes to the differentiation of cells [33,34]. Previous reports have revealed that autophagy is involved in the atresia of follicles and the regression of CLs in mammals in a reproductive system [35,36]. However, as an important hormone-dependent gland, there remain gaps in the underlying mechanism for regulation and contribution of autophagy to progesterone production during the luteal development of pregnant ovaries in mammals. Therefore, the present study utilized a rat model of pregnancy to investigate the variation of autophagy levels and found the levels of autophagy-related proteins were increased obviously from day 10 to day 16 and then compromised to a relatively low level on day 21 after pregnancy, suggesting autophagy may play an important role during the luteal development of pregnant ovaries in vivo.

The maintenance of a high autophagy level is generally linked to specific physiological functions. Previous reports have demonstrated the involvement of autophagy in regulating functional redetermination of cells in a reproductive system, including the atresia of follicle, the regression of CL, and the transition of oocyte to embryo [36,37,38]. In male, the leydig cells in tests showed similar functions with the CL in female, where high levels of autophagy were also observed and of which the maintenance was essential for its steroid secretion [7]. However, the involvement and physiological functions of autophagy in pregnant CLs remain elusive. Interestingly, our present study found the variation of autophagy was synchronized with the change of serum progesterone levels during the luteal development of pregnant ovaries, suggesting a possible role of autophagy in progesterone synthesis. Further investigations showed the treatment of an autophagy inhibitor 3-MA diminished the elevation of serum progesterone levels on day 16 while no obvious changes were identified on day 10 and day 21. Furthermore, the results of chloroquine (CQ) treatment showed similar effects on serum progesterone levels, indicating autophagy as a pivotal regulator in progesterone production (Supplementary Figure S1). To corroborate this finding, the present study knockdowned the expression of an initiator of autophagy induction Beclin1 by siRNA transfection or 3-MA treatment to curtail autophagy levels, and it was found progesterone secretion was markedly compromised in these cultured luteal cells while an mTOR inhibitor rapamycin increased progesterone production possibly by promoting autophagy in cultured luteal cells. It is noteworthy that we performed different cell treatment schedules mainly given that the production of progesterone is not an acute program, as the previous investigations showed that even a gonadotropin treatment was unable to significantly affect progesterone levels with acute incubation for 3 h [23]. These findings are consistent with previous investigations showing autophagy deficiency is linked to the compromise of steroidogenic ability [7]. Nevertheless, our present data demonstrated a regulatory role of autophagy in progesterone synthesis, which may be an essential factor in influencing rat pregnancy.

The Akt/mTOR pathway is known to be a controller of the autophagic mechanism in mammalian cells, which is also involved in autophagy induction and steroid secretion in steroidogenic cells [7]. However, the contribution of Akt/mTOR signaling to autophagy induction in luteal cells of pregnant ovaries still remained unclear. Therefore, the present study examined the involvement of the Akt/mTOR pathway in autophagy induction during the luteal development of pregnant ovaries in vivo and found Akt/mTOR signaling was dramatically decreased on day 16 and maintained at a low level on day 21 after pregnancy. To further confirm the effect of mTOR signaling on autophagy induction in luteal cells, an inhibitor of mTOR rapamycin was used to treat isolated luteal cells from rat ovaries on day 16 after pregnancy and found autophagy was obviously induced in a time-dependent manner in vitro. These results clearly demonstrated that autophagy is regulated through Akt/mTOR signaling, participating in the production of progesterone during the luteal development of pregnant rats.

In steroidogenic cells, two main factors influenced the synthesis of steroid, constant supply of cholesterol and activation of mitochondria. There are three potential sources of cholesterol pools that could be mobilized for luteal steroidogenesis, de novo synthesis, hydrolysis of stored cholesterol esters, and exogenous lipoproteins [39], although high- and low-density lipoproteins (HDLs and LDLs) from plasma are two main kinds of cholesterol for CLs. The utilization of stored lipid, especially cholesterol, is also crucial for steroidogenic cells to produce androgens, glucocorticoids, mineralocorticoids, estrogens, and progesterone [24]. Formation of lipid droplets is a ubiquitous practice in steroidogenic cells and used for raw material preparation of steroidogenesis [40,41,42]. The role of autophagy in catabolism of lipid droplets has been described in many reports [43], but autophagy is also suggested to play a positive role in regulating lipid droplets growth and accumulation in some tissues, such as adipose tissues of obese and diabetic individuals [44]. Knockdown of Beclin1 in cultured 3T3-L1 adipocytes also curtailed lipid storage [45]. A significant decrease of serum progesterone levels was observed during pregnancy in a Beclin1 conditional knockout murine model, which was murine resulting from the significant decrease of lipid droplet storage [24]. Therefore, the present study examined whether autophagy was also devoted to progesterone synthesis by modulating lipid droplets formation in CLs and found the intensity of Oil Red O staining was stronger on day 16 than that on day 10 and maintained at a basal level on day 21 after pregnancy, which was analogous to TEM observation. However, the 3-MA treatment exacerbated the storage of lipid droplets in luteal cells, as evidenced by decreased Oil Red O staining intensity and salient shrinkage of droplet volumes. These results implied that autophagy may contribute to progesterone synthesis by modulating cholesterol or lipid storage during the luteal development of pregnant rats. However, as we did not conduct experiments to further verify the role of lipid droplet in progesterone production, there also exists a gap to determine how the content of lipid droplet affects progesterone levels.

Mitochondria are another factor in influencing progesterone synthesis. A compelling demand of progesterone supply at the noon of the CL status requires the full-load operation of mitochondria [8], which may accelerate the aging of an organelle and also impair the balance of mitochondrial catabolism. It is well-known that mitochondria are also the primary source of many endogenous toxic agents, among which the increase of cytochrome C leakage generally occurs with mitochondrial dysfunction, which in turn skews homeostasis of cells [46]. Autophagy is an available mechanism responsible for mitochondrial quality control whereupon protecting the highly regulated mitochondrial status from disturbing [17]. A previous investigation has revealed an increase of endogenous toxin levels during functional regression of rat CLs and suggested a potential role in early luteal regression [8]. In the present study, an increase in intracellular cytochrome C and a decrease in mitochondrial number were observed on day 21 compared with day 16. Further investigations suggested that 3-MA treatment hindered the curtailment of mitochondria in CLs on day 21 whereas the maintenance of mitochondria promoted cytochrome C release compared with in controls. A similar mechanism could also be found in other types of cells [45,46,47,48], implying autophagy may be involved in the elimination of mitochondria during the initiation of functional regression in CLs, which may be a ubiquitous mechanism for mammalian cells to inhibit release of cytochrome C and advanced activation of cell apoptosis. It also needs to be noticed that gene knockout experiments are further required to determine detailed contribution of autophagy in this luteal phase stage, as the treatment of 3-MA is not a sufficient evidence to determine the autophagic role.

## 5. Conclusions

Together, the present study demonstrated the expression of autophagy-related proteins in CLs was regulated by Akt/mTOR signaling and the contribution of autophagy to progesterone production was regulated by, at least in part, modulating the pool of lipid droplets in luteal cells during the luteal development of pregnant rats. Furthermore, based on present evidences, we inferred that mitophagy was also involved in the control of the mitochondrial pool and quality during the initiation of luteal regression in pregnant rats. Therefore, the attenuation of autophagy will hamper the orchestration of luteal homeostasis, and our present findings will shed light on the role of autophagy during the luteal development of pregnant mammals in vivo.

## Supplementary Materials

The following are available online at https://www.mdpi.com/2073-4409/9/1/71/s1, Figure S1: Changes of serum progesterone levels during the luteal development of pregnant rats treated with chloroquine, Table S1: Antibody informations for western blotting.
