# Supplementary material for: Autophagy Attenuation Hampers Progesterone Synthesis during the Development of Pregnant Corpus Luteum
Source: Cells. 2019 Dec 27;9(1):71. doi: 10.3390/cells9010071 (PMC7016947; doi:10.3390/cells9010071)
Supplement: Supplementary file 1 [file cells-09-00071-s001.pdf]

cells

Supplementary Materials

Autophagy Attenuation Hampers Progesterone Synthesis during the Development of Pregnant Corpus Luteum

Zonghao Tang [1,2,†], Zhenghong Zhang [1,†], Hong Zhang [1,†], Yuhua Wang [1], Yan Zhang [1], Jiuhua Zhao [1], Hongqin Yang [1,*] and Zhengchao Wang [1,*]

[1] Provincial Key Laboratory for Developmental Biology and Neurosciences, Key Laboratory of Optoelectronic Science and Technology for Medicine of Ministry of Education, College of Life Sciences, Fujian Normal University, Fuzhou 350007, China; tangzonghao@163.com (Z.T.); zhangzh@fjnu.edu.cn (Z.Z.); ZhHong0898@163.com (H.Z.); yuhwang@fjnu.edu.cn (Y.W.); yanzhang970118@sina.com (Y.Z.); zhao9hua@126.com (J.Z.)

[2] Drug Discovery Research Center, Southwest Medical University, Luzhou 646000, China

* Correspondence: hqyang@fjnu.edu.cn (H.Y.); zcwang@fjnu.edu.cn (Z.W.)

† Authors have equal contribution.

Received: 11 October 2019; Accepted: 24 December 2019; Published: date

1. Supplementary Figure 1

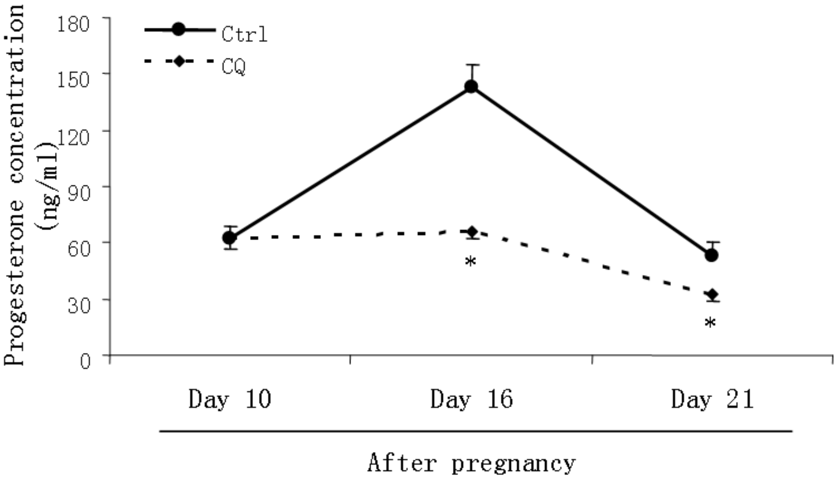

Supplementary Figure 1. Changes of serum progesterone levels during the luteal development of pregnant rats treated with chloroquine. In order to further determine the possible roles of autophagy, chloroquine (CQ, i.p. 20 mg/kg body weight, Sigma-Aldrich) was injected according to the method described by Choi et al. Briefly, chloroquine was dissolved with sterile saline and then pregnant rats were consecutively treated for 5 days (i.p.) before samples collection, saline was served as the control/vehicle. All pregnant rats were executed at three designed time points, including Day 10 when progesterone surging, Day 16 when CL status or functions at noon and Day 21 when circulatory progesterone level dramatically decreased. Each value represents the mean ± SE, n = 3. One-way analysis of variance (ANOVA) was used to analyze the data. *: $p < 0.05$, vs. Ctrl. CQ: chloroquine, an autophagy inhibitor.

2. Supplementary Table 1

Supplementary Table 1. Antibody informations for western blotting.

Antibody Name	Company and City	Catalogue Number	Dilution Degree
LC-3	Abcam, Cambridge, MA, USA	ab128025	1:1000
Beclin1	Protein Tech Group, Wuhan, China	11306-1-AP	1:2000
β-actin	Protein Tech Group, Wuhan, China	20536-1-AP	1:5000
LAMP-2	Protein Tech Group, Wuhan, China	27823-1-AP	1:500
p62	Abcam, Cambridge, MA, USA	ab109012	1:1000
pAkt (S473)	Cell Signaling Technology, Boston, MA, USA	4060	1:1000
Akt	Cell Signaling Technology, Boston, MA, USA	4691	1:1000
mTOR	Protein Tech Group, Wuhan, China	20657-1-AP	1:500
p-p70S6K (Thr389)	Cell Signaling Technology, Boston, MA, USA	97596	1:1000
COXIV	Protein Tech Group, Wuhan, China	11242-1-AP	1:2000
VDAC1	Protein Tech Group, Wuhan, China	10866-1-AP	1:500
cytochrome C	Protein Tech Group, Wuhan, China	66264-1-Ig	1:2000
HIF-1α	Santa Cruz Biotechnology, Dallas, TX, US	sc-13515	1:500
BNIP3	Abcam, Cambridge, MA, USA	ab10433	1:1000
PINK1	Affinity Biosciences, Cincinnati, OH USA	DF7742	1:1000
anti-Mouse IgG	Beyotime Institute of Biotechnology, Haimen, China	A7028	1:5000
anti-Rabbit IgG	Beyotime Institute of Biotechnology, Haimen, China	A7016	1:5000
